# Are lipid-lowering drugs harmful to cognitive performance?: A Mendelian randomization study

**DOI:** 10.1097/MD.0000000000044260

**Published:** 2025-09-05

**Authors:** Lijun Han, Chang Su, Ruonan Lian, Junming Han, Xiude Fan, Ling Gao

**Affiliations:** a Key Laboratory of Endocrine Glucose & Lipids Metabolism and Brain Aging, Ministry of Education; Department of Endocrinology, Shandong Provincial Hospital Affiliated to Shandong First Medical University, Jinan, Shandong Province, China; b Shandong Clinical Research Center of Diabetes and Metabolic Diseases, Jinan, Shandong Province, China; c Shandong Institute of Endocrine and Metabolic Diseases, Jinan, Shandong Province, China; d “Chuangxin China” Innovation Base of Stem Cell and Gene Therapy for Endocrine Metabolic Diseases, Jinan, Shandong Province, China; e Shandong Engineering Laboratory of Prevention and Control for Endocrine and Metabolic Diseases, Jinan, Shandong Province, China; f Shandong Engineering Research Center of Stem Cell and Gene Therapy for Endocrine and Metabolic Diseases, Jinan, Shandong Province, China.

**Keywords:** cognitive performance, ezetimibe, Mendelian randomization, PSCK9 inhibitors, statins

## Abstract

Cardiovascular disease is the leading global cause of mortality, affecting the development of cognitive impairment in the elderly. Lipid-lowering drugs are commonly used to manage cardiovascular disease risk, but their effects on cognitive performance have produced conflicting results in previous research. To better guide the selective decision-making and application of lipid-lowering drugs, this study aims to determine the causal relationship between lipid-lowering drugs and cognitive performance through Mendelian randomization. We conducted a 2-sample Mendelian randomization study based on summary statistics from genome-wide association studies for lipid-lowering drugs and cognitive performance. Single-nucleotide polymorphisms significantly related to low-density lipoprotein cholesterol or triglycerides and associated with drug target genes served as proxies for statins, ezetimibe, proprotein convertase subtilisin/kexin type 9 (PCSK9) inhibitors, fibrates, etc. Genetically predicted statins (*β* = −0.094, 95% confidence interval −0.138 to −0.049, *P* < .001) and ezetimibe (*β* = −0.319, 95% confidence interval −0.408 to −0.231, *P* < .001) had adverse effects on cognitive performance, while other drugs, including PCSK9 inhibitors and fibrates, etc showed no significant impact. This study suggests that statins and ezetimibe may have adverse effects on cognition, while PCSK9 inhibitors and fibrates appear to have no such effect, which may help clinicians make more informed prescription decisions. These findings should be interpreted carefully and require further validation by long-term follow-up clinical studies.

## 1. Introduction

Cardiovascular disease (CVD) is still the leading cause of worldwide mortality, causing more than 18.6 million deaths each year and affecting the development of cognitive impairment in the elderly.^[1,2]^ Although lifestyle changes can reduce CVD risk, lipid-lowering drugs such as statins, proprotein convertase subtilisin/kexin type 9 (PCSK9) inhibitors, ezetimibe, and fibrates remain important tools for managing cardiovascular health in high-risk patients and have been widely used in clinical practice. The adverse event reporting system (FAERS) of the United States Food and Drug Administration (FDA) reported the adverse effects of lipid-lowering drugs on cognition. Previous studies have reported varied associations between lipid-lowering drugs and cognitive performance, which seem to depend on the type of drugs. Observational studies are still inconclusive regarding statins; some suggest an association with cognitive impairment, while others suggest improvement in cognitive function. Rosoff et al performed Mendelian randomization (MR) analyses indicating that HMG-CoA reductase inhibitors are associated with adverse cognitive performance.^[3]^ Moreover, only a few large-scale randomized controlled trials (RCTs) of statins are available.^[4–11]^ For PCSK9 inhibitors, genetic studies have identified a role of PCSK9 in central nervous system disease pathology, including Alzheimer disease and neuropsychiatric disorders.^[12,13]^ However, RCT and meta-analysis have shown no association with cognitive performance.^[14–16]^ In addition, the current MR analysis failed to get exact evidence about the relationship between them.^[3,17]^ For ezetimibe, fibrates, and other lipid-lowering drugs, there are no related clinical studies regarding their impact on cognitive performance.

However, observational studies are vulnerable to potential bias, such as confounding and reverse causation. Additionally, there is a lack of large-scale and long-term RCTs to evaluate the effects of lipid-lowering drugs on cognitive performance. Moreover, since FDA reports do not provide proof of causality, we cannot infer causation from observed associations in the database. Therefore, we introduced MR, a genetic epidemiological method. MR emulates the random distribution of treatments in clinical trials and is based on the natural and random distribution of genetic variants during meiosis.

As CVD affects the development of cognitive impairment, understanding whether lipid-lowering drugs impact cognitive performance will enable clinicians to make more informed prescribing decisions. This study evaluated the adverse reactions of lipid-lowering drugs on cognitive performance by analyzing the electronic records from FAERS and further applied MR to determine the causal relationship between lipid-lowering drugs and cognitive performance including statins, PCSK9 inhibitors, ezetimibe, fibrates, and other lipid-lowering drugs.

## 2. Materials and methods

### 2.1. Study design and data sources

Established by the US FDA, the FAERS is a key source for worldwide pharmacovigilance, which our study utilized to investigate the correlation between drug use and adverse events from January 2004 to June 2022 via the openFDA platform. We conducted a disproportionality analysis using the proportional reporting ratio (PRR) and reporting odds ratio (ROR). Signals were considered significant if the PRR ≥ 2, *χ*^2^ ≥ 4, and when the lower limit of ROR > 1 with confidence intervals (CIs) did not include 1.^[18,19]^ All analyses were conducted using the R Statistics Software platform (R Foundation for Statistical Computing, Vienna, Austria).

To address potential confounding factors in our analysis, we adopted MR which uses appropriate genetic variants as instrumental variables (IVs) to represent the exposure factors under investigation. By measuring the correlation between genetic variation and exposure, genetic variation and outcome, we can infer the relationship between exposure and outcome.^[20]^ On this basis, compared to conventional MR, our instruments were based on drug target genes to proxy drug effects, rather than genetic variants from all over the genome.^[21]^ Our MR analysis utilized a 2-sample design with summary data from genome-wide association studies (GWAS) resources, which had already obtained ethical review board approvals. In addition, since coronary heart disease is a primary indication of these drugs, it was used as a positive control to evaluate the effectiveness of IVs.^[3]^

This study used summary statistics from the GWAS database, with data from the Global Lipid Genetics Federation to estimate the association between genetic variation and the reduction of low-density lipoprotein cholesterol (LDL-C) and triglycerides (TG) caused by lipid-lowering drugs. The analysis included lipid profiles (high-density lipoprotein cholesterol, LDL-C, TG, and total cholesterol) from over 188,000 individuals across 60 studies.^[22]^ Cognitive performance as an outcome was measured by the mean standardized test score from a weighted meta-analysis (N = 257,841). This analysis incorporated the first principal component derived from at least 3 neuropsychological tests from the Cognitive Genomics Consortium, as well as scores from a verbal-numerical reasoning test which contains 13 logic and reasoning questions with a 2-minute time limit obtained from the UK Biobank.^[23,24]^ In addition, data from 22,233 patients with coronary heart disease and 64,762 European controls was sourced from CARDIoGRAMplusC4D^[25]^ (Table S1, Supplemental Digital Content, https://links.lww.com/MD/P878).

### 2.2. Statistical methods and sensitivity analyses

To identify target genes for lipid-lowering drugs, we utilized DrugBank and referenced the Anatomical, Therapeutic, and Chemical classification system, published by the World Health Organization, to extract comprehensive information including drug names, DrugBank IDs, and target genes. As of 2023, 12 lipid-lowering drugs targeting 34 genes were identified (Table S2, Supplemental Digital Content, https://links.lww.com/MD/P878). These include 3-hydroxy-3-methyl-glutaryl-coenzyme A reductase for statins, Niemann-Pick C1-Like 1 for ezetimibe, and PCSK9 for PCSK9 inhibitors among LDL-C-reducing drugs, and peroxisome proliferator-activated receptor gamma, angiopoietin-like protein 3 (ANGPTL3), and lipoprotein lipase (LPL) for TG-reducing drugs.

MR framework is based on 3 key assumptions: (a) correlation hypothesis: genetic variation as an IV is strongly correlated with the exposure factors of the study; (b) independence hypothesis: IVs are independent of any confounding factors that affect exposure factors and results; (c) exclusivity hypothesis: IVs only affect the results through their influence on exposure factors, but not through any other means.

**Figure 1.**
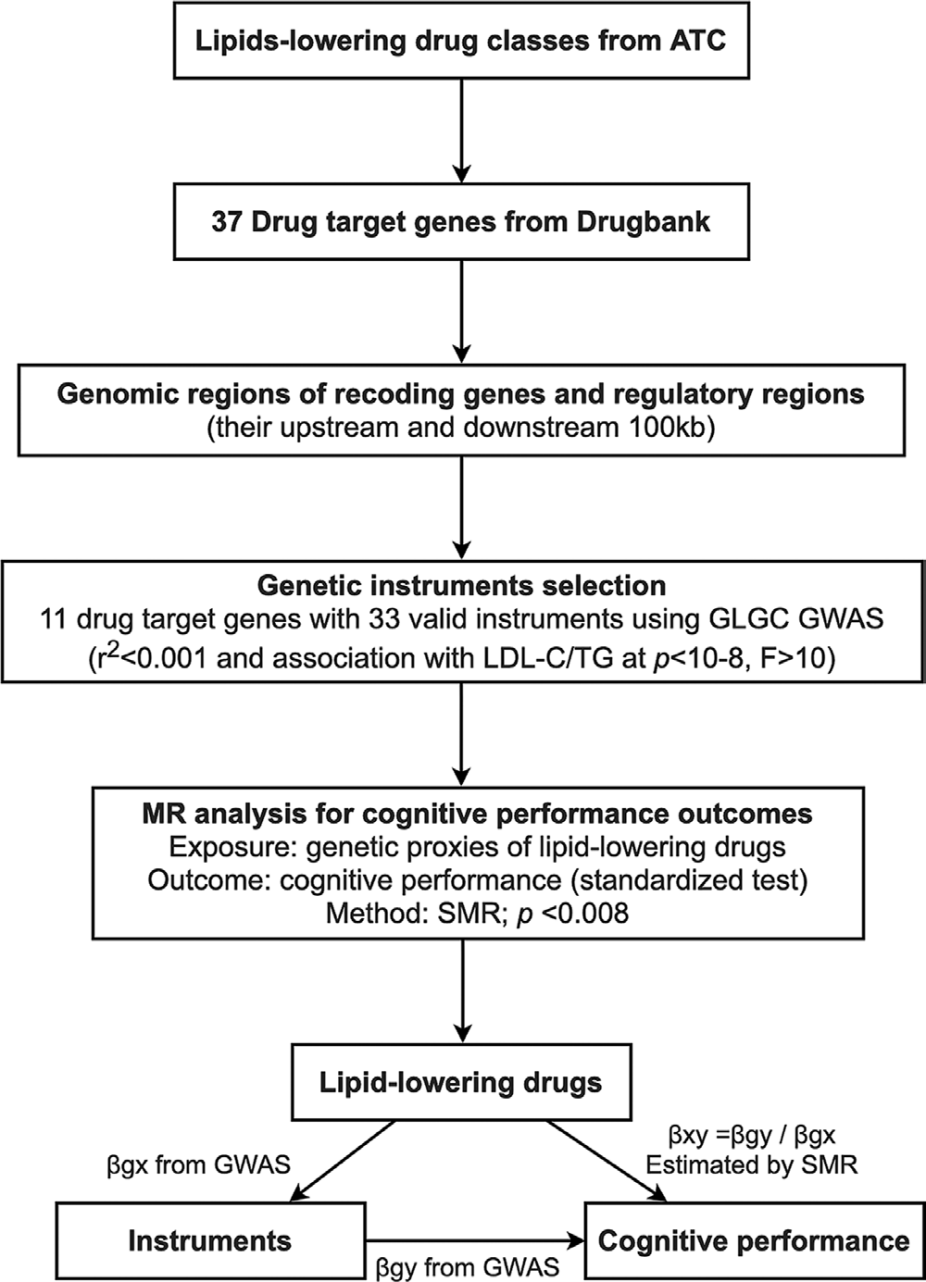
Summary of study design. ATC = Anatomical, Therapeutic, and Chemical, GLGC = Global Lipid Genetics Federation, GWAS = genome-wide association study, LDL-C = low-density lipoprotein cholesterol, MR = Mendelian randomization, SMR = summary-based MR, TG = triglycerides, *β*_gx_ = the association between genetic variation and exposure, *β*_gy_ = the association between genetic variation and outcome, *β*_xy_ = the estimated effect of the exposure on the outcome.

To proxy lipid-lowering drugs, we used single nucleotide polymorphisms (SNPs) located within 100 kilobases of these target genes and significantly related to LDL-C and TG (*P* < 5 × 10^−8^) as IVs because the decrease of LDL-C and TG in circulation is the primary effect of lipid-lowering drugs. SNPs with *F* statistics >10 were selected to minimize weak instrument deviations. To ensure independence and avoid redundancy, SNPs with linkage disequilibrium *r*^2^ > 0.001 (European population reference) were excluded. In total, 29 and 4 SNPs were chosen to evaluate the effects of genetic proxies for lipid-lowering drugs on cognitive performance (Fig. 1; Table S3, Supplemental Digital Content, https://links.lww.com/MD/P878). All data analyses were carried out using 2-sample MR through the inverse variance weighting method. Bonferroni adjustment was applied to 6 drug target genes to correct for multiple comparisons, with *P* < .008 considered statistically significant. All analyses were performed with the Mendelian randomization package in R Statistics Software platform version 4.1.2 (R Foundation for Statistical Computing, Vienna, Austria).

As part of our sensitivity analysis, we also applied other methods available in the 2-sample MR 0.5.5 R package to control potential pleiotropy. Our analysis used the intercept from MR-Egger regression, which is considered statistically significant at *P* < .05. This method can provide reliable estimates even if all selected SNPs are invalid instruments.^[26]^ Besides, the weighted median method was used as a confirmatory MR analysis, which can yield a fair estimate provided that over 50% of the SNPs are valid IVs.^[27]^ This method has shown greater robustness than MR-Egger regression when IVs assumptions are violated and has significantly enhanced accuracy. Heterogeneity across MR estimates was assessed with the Cochran *Q* test; results with *P* < .05 were deemed statistically significant.^[28]^ To further ensure the robustness of our results, a leave-one-out analysis was performed to identify any single SNPs that might excessively influence the MR estimation.

## 3. Results

In total, 612,696 reports related to lipid-lowering drugs were retrieved from the FAERS database, of which 12,791 reports (2.088%) were associated with adverse cognitive performance. Significant signals indicated by ROR included 5 statins (atorvastatin ROR 1.42, 95% confidence interval [CI] 1.381–1.472; rosuvastatin ROR 1.228, 95% CI 1.165–1.294; pravastatin ROR 1.317, 95% CI 1.229–1.413; lovastatin ROR 1.519, 95% CI 1.357–1.700; simvastatin ROR 1.371, 95% CI 1.321–1.422). Ezetimibe also exhibited a significant signal (ROR 1.107, 95% CI 1.029–1.191), as did 2 fibrates (fenofibrate ROR 1.107, 95% CI 1.029–1.191; gemfibrozil ROR 1.417, 95% CI 1.213–1.655). Cerivastatin of statins (PRR 3.335, *χ*^2^ 17.659; ROR 3.481, 95% CI 1.942–6.237) also had a significant signal but it had already been withdrawn from the market in 2001. Complete results are presented in Table S4, Supplemental Digital Content, https://links.lww.com/MD/P878.

**Figure 2.**
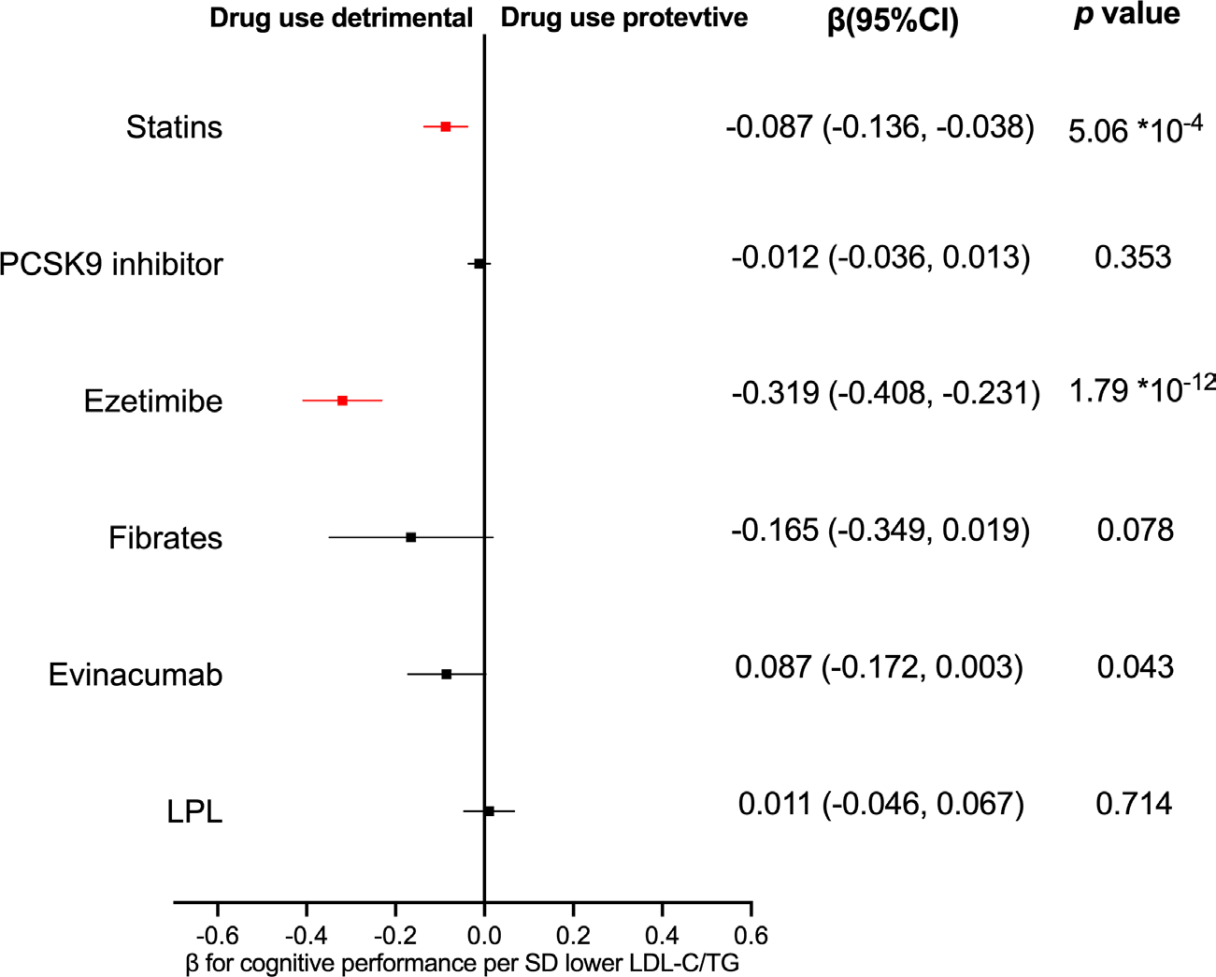
Mendelian randomization associations between genetic proxies for lipid-lowering drugs and cognitive performance (standardized test). IVW-MR method was used to assess the association. CI = confidence interval, IVW = inverse variance weighted, LDL-C = low-density lipoprotein cholesterol, LPL = lipoprotein lipase, MR = Mendelian randomization, PCSK9 = proprotein convertase subtilisin/kexin type 9, SD = standard deviation, TG = triglycerides.

**Figure 3.**
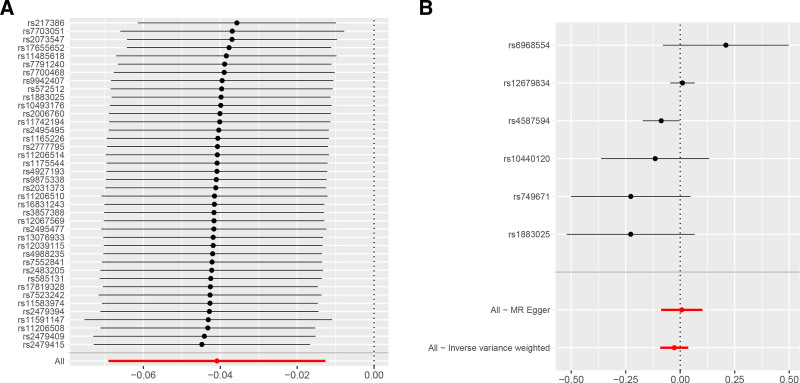
MR leave-one-out sensitivity analysis for “Lipids-Lowering Drugs” on “Cognitive performance.” (A) MR leave-one-out sensitivity analysis for “LDLC-Lowering Drugs” on “Cognitive performance” (B) MR leave-one-out sensitivity analysis for “TG-Lowering Drugs” on “Cognitive performance.” LDLC = low-density lipoprotein cholesterol, MR = Mendelian randomization, TG = triglycerides.

Consistently, our MR study revealed that genetically predicted statins (*β* = −0.087, 95% CI −0.136 to −0.038, *P* < .001) and ezetimibe (*β* = −0.319, 95% CI −0.408 to −0.231, *P* < .001) were harmful to cognitive performance score after correcting for multiple testing (Fig. 2). These results were consistent across other models, including weighted median, weighted mode, and Mendelian Randomization Pleiotropy RESidual Sum and Outlier (MR-PRESSO). The CIs for statins and ezetimibe were narrow and excluded the null effect, and the beta coefficients indicated that drug use was detrimental. In contrast, PCSK9 inhibitors, fibrates, ANGPTL, and LPL were not statistically significant in cognitive performance. These results were consistent when analyzed using alternative models. The CIs for PCSK9 inhibitors and LPL were narrow and included a null effect, and the beta coefficients were very close to zero, indicating no significant effect. However, the CIs for fibrates and ANGPTL3 were wide and included the null, suggesting uncertainty in these estimates. Complete results are presented in Table 1. Overall, no evidence of heterogeneity or horizontal pleiotropy was observed, as could be seen in the nonsignificant Cochran *Q* test and the MR-Egger intercept which was very close to zero. The MR-Egger intercept represents the average pleiotropic effect of the genetic variants included in the analysis. When the intercept value differs significantly from 0, the conventional MR estimate will be biased (either directional pleiotropy is present, or the MR assumption is violated). Furthermore, the leave-one-out analysis confirmed that no individual SNPs had a substantial impact on the result, as all data points were positioned on the right side of 0 (Fig. 3).

**Table 1:** Two-sample Mendelian randomization model estimates for the effects of modulating lipid-lowering drug targets on cognitive performance (standardized test).

	Gene	Method	*β* (95% CI)	*P* *	Q_pval	nSNPs
HMG-CoA reductase inhibitor	HMGCR	IVW	**−0.087 (−0.136, −0.038**)	**5.06E−04**	.885	6
		Weighted median	**−0.092 (−0.151, −0.033**)	**.002**		
		Weighted mode	**−0.096 (−0.165, −0.028**)	**.006**		
		MR-Egger	−0.196 (−0.475, 0.083)	.169		
		Intercept	0.006 (−0.010, 0.022)	.438		
		MR-PRESSO	**−0.087**	**.002**		
PCSK9 inhibitor	PCSK9	IVW	−0.012 (−0.036, 0.013)	.353	.572	19
		Weighted median	−0.030 (−0.064, 0.004)	.083		
		Weighted mode	−0.027 (−0.064, 0.010)	.125		
		MR-Egger	−0.036 (−0.081, 0.009)	.119		
		Intercept	0.002 (−0.001, 0.005)	.209		
		MR-PRESSO	−0.087	.002		
Ezetimibe	NPC1L1	IVW	**−0.319 (−0.408, −0.231**)	**1.79E−12**	.666	4
		Weighted median	**−0.313 (−0.428, −0.199**)	**7.86E−08**		
		Weighted mode	**−0.288 (−0.434, −0.141**)	**1.16E−04**		
		MR-Egger	−0.047 (−0.491, 0.397)	.835		
		Intercept	−0.011 (−0.027, 0.006)	.220		
		MR-PRESSO	−**0.319**	**.002**		
Fibrates	PPARG	IVW	−0.115 (−0.362, 0.133)	.364		1
	MMP25	IVW	−0.227 (−0.501, 0.047)	.105		1
ANGPTL3	ANGPTL3	IVW	−0.087 (−0.172, 0.003)	.043		1
LPL	LPL	IVW	0.011 (−0.046, 0.067)	.714		1

Significant *P* values are in bold.

ANGPTL3 = angiopoietin-like protein 3, CI = confidence interval, HMGCR = 3-hydroxy-3-methyl-glutaryl-coenzyme A reductase, IVW = inverse-variance weighted, LPL = lipoprotein lipase, MR-PRESSO = Mendelian Randomization Pleiotropy RESidual Sum and Outlier, NPC1L1 = Niemann-Pick C1-Like 1, PCSK9 = proprotein convertase subtilisin/kexin type 9, PPARG = peroxisome proliferator-activated receptor gamma, Q_pval = *P* value of heterogeneity test statistic (Cochran *Q*), SNP = single nucleotide polymorphism.

* Multiple testing is correcting *P*-value equal to (0.05/6 = 0.008).

**Figure 4.**
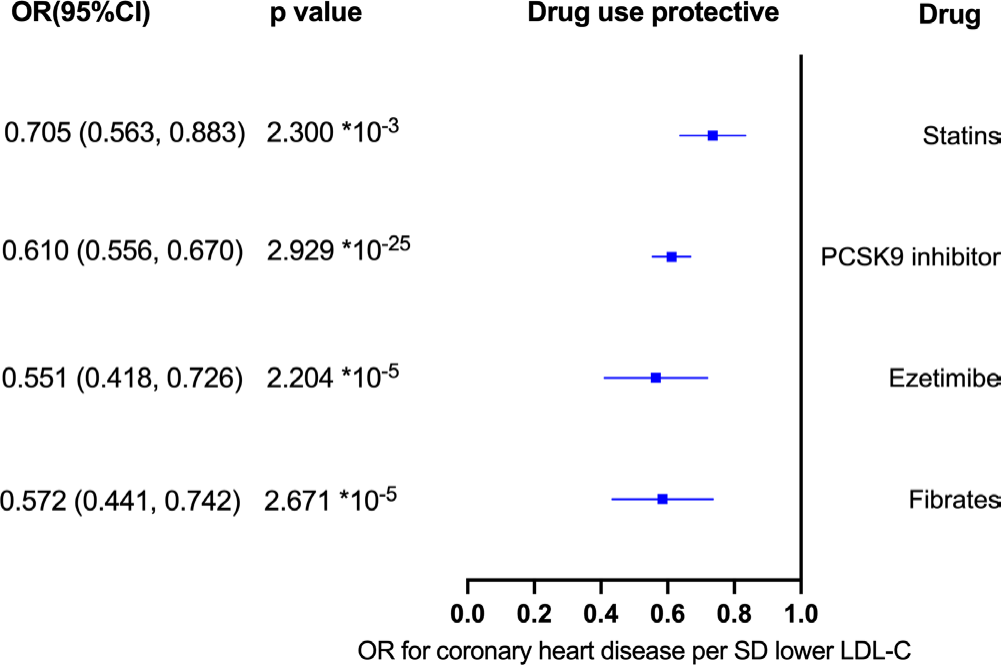
Mendelian randomization associations between genetic proxies for lipid-lowering drugs and coronary heart disease. IVW-MR method was used to assess the association. CI = confidence interval, IVW = inverse variance weighted, LDL-C = low-density lipoprotein cholesterol, MR = Mendelian randomization, OR = odds ratio, PCSK9 = proprotein convertase subtilisin/kexin type 9, SD = standard deviation.

As expected, the positive control showed that statins (odds ratio [OR] = 0.705, 95% CI 0.563–0.883, *P* = 2.300 × 10^−3^), ezetimibe (OR = 0.551, 95% CI 0.418–0.726, *P* = 2.204 × 10^−5^), PSCK9 inhibitors (OR = 0.610, 95% CI 0.556–0.670, *P* = 2.929 × 10^−25^), and fibrates (OR = 0.572, 95% CI 0.441–0.742, *P* = 2.671 × 10^−5^] significantly decreased the risk of coronary heart disease (Fig. 4), which supported the validity of genetic instruments.

## 4. Discussion

From the FAERS reports, although quite rare (2.088%) compared to all adverse event reports, lipid-lowering drugs can cause adverse cognitive performance, with atorvastatin, rosuvastatin, pravastatin, simvastatin, ezetimibe, and fenofibrate accounting for the majority of reports. In our study, genetically predicted statins and ezetimibe were observed to have an adverse lifelong effect on cognitive performance. Conversely, other drugs including PCSK9 inhibitors, fibrates, ANGPTL3, and LPL demonstrated no significant impact on cognitive performance. Our results were consistent across various MR methods (inverse variance weighting, weighted median, weighted mode, and MR-PRESSO) which made different assumptions about the presence of pleiotropy, thus avoiding potential false positives that could arise from a single-method approach, thereby strengthening the causal inference of our study.

The exact cause of statin-induced cognitive dysfunction is unclear. However, animal studies have suggested that this could be related to the reduction of cholesterol synthesis in oligodendrocytes, leading to inhibited central nervous system myelination, which in turn may contribute to cognitive deficits. Furthermore, the brain levels of statins are influenced by their lipophilicity and the rate at which they are eliminated from the brain.^[29]^ Despite these concerns, it is critical to recognize that there is plenty of evidence supporting the overall benefits of statins outweigh the risks.^[30]^ These insights are crucial for clinicians to make more informed decisions when prescribing drugs to treat CVD.

Previous research has shown that when ezetimibe is added to various statin drugs, it can reduce C-reactive protein levels, indicating its anti-inflammatory effects.^[31]^ Additionally, it has been observed to ameliorate steatohepatitis via autophagy induction.^[32]^ While earlier studies focused on its anti-inflammatory and liver-related properties, our research extends to its adverse cognitive effects, expanding the findings reported in existing studies and highlighting the need for a comprehensive understanding of its various actions and potential consequences on health.

The screening of FDA drug adverse reactions revealed records of adverse drug reactions associated with fenofibrate. However, our study demonstrated no association between fibrates and cognitive performance. The literature on the relationship between fibrates and cognition is limited, with one study suggesting that fenofibrate may have receptor-dependent neuroprotective effects, attributed to its regulation of oxidative stress and inflammation.^[33]^ These findings collectively indicate that there is limited evidence directly linking fibrates to cognitive function, despite the presence of adverse drug reaction records, emphasizing the need for further research in this area.

Besides, there have been reports of PCSK9 inhibitors affecting cognition,^[34]^ however, the vast majority of RCTs and meta-analyses have shown no association.^[14,15,35]^ Even though more future clinical studies are necessary to determine whether PCSK9 expression affects cognition, our results align with existing short-term data, indicating no adverse impact on cognitive performance with PCSK9 inhibitors. These results help to alleviate ongoing concerns regarding the neurocognitive safety of PCSK9 inhibitors.

However, the study has several limitations. First of all, genetic variation explains only a fraction of the exposure variance, meaning that MR may not be as accurate as conventional observational studies. Although there are fewer confounding factors in MR, we cannot rule out the possibility of inaccuracies contributing to empty correlation. Second, weak tools may be biased towards zero, however, we only use genetic variations with *F* statistics >10. Third, even though the results of diverse MR methods are consistent and various sensitivity analyses have been carried out, residual pleiotropy cannot be completely ruled out. However, because the MR-Egger intercept is at the center of the origin, this impact is likely to be very small. Fourth, the MR study focuses on lifetime associations, which may not directly correspond to the short-term effects of taking lipid-lowering drugs. Therefore, our findings are more pertinent in evaluating the direction rather than the degree of associations. Fifth, since the GWAS data used predominantly originate from the European ancestry population, these findings should be carefully interpreted when extended to other populations. Considering the multiple effects of lipid-lowering drugs and their correlation with various non-lipid markers, it is difficult to determine appropriate negative control outcomes.

## 5. Conclusion

In summary, this study assessed the potential effects of various types of lipid-lowering drugs on cognitive performance. It suggests that statins and ezetimibe may have adverse effects on cognition, while PCSK9 inhibitors and fibrates appear to have no such effect, which may help clinicians make more informed prescription decisions. Given the limitations of MR, these findings should be interpreted carefully and further validated by long-term follow-up clinical studies.

## Acknowledgments

We are grateful for all the GWASs making the summary-level data publicly available.

## Author contributions

**Conceptualization:** Lijun Han, Ling Gao.

**Investigation:** Lijun Han, Chang Su, Ruonan Lian.

**Methodology:** Lijun Han, Xiude Fan.

**Project administration:** Ling Gao.

**Software:** Lijun Han, Chang Su, Ruonan Lian, Junming Han.

**Supervision:** Ling Gao.

**Validation:** Lijun Han, Chang Su, Ruonan Lian.

**Visualization:** Xiude Fan.

**Writing – original draft:** Lijun Han.

**Writing – review & editing:** Ling Gao.

## Supplementary Material


